# Progress on long non-coding RNAs in calcific aortic valve disease

**DOI:** 10.3389/fcvm.2025.1522544

**Published:** 2025-01-17

**Authors:** Yan Shen, Jiahui Li, Zehao Zhao, Xiaomin Chen

**Affiliations:** ^1^ Department of Cardiology, The First Affiliated Hospital of Ningbo University, Ningbo, China; ^2^ Department of Cardiology, The Second Affiliated Hospital of Zhejiang University, Hangzhou, China

**Keywords:** calcific aortic valve disease, long non-coding RNA, valve interstitial cells, osteogenic differentiation, mechanisms

## Abstract

Calcific aortic valve disease (CAVD) is a common cardiovascular condition in the elderly population. The aortic valve, influenced by factors such as endothelial dysfunction, inflammation, oxidative stress, lipid metabolism disorders, calcium deposition, and extracellular matrix remodeling, undergoes fibrosis and calcification, ultimately leading to stenosis. In recent years, long non-coding RNAs (lncRNAs) have emerged as significant regulators of gene expression, playing crucial roles in the occurrence and progression of various diseases. Research has shown that lncRNAs participate in the pathological process underlying CAVD by regulating osteogenic differentiation and inflammatory response of valve interstitial cells. Specifically, lncRNAs, such as H19, MALAT1, and TUG1, are closely associated with CAVD. Some lncRNAs can act as miRNA sponges, form complex regulatory networks, and modulate the expression of calcification-related genes. In brief, this review discusses the mechanisms and potential therapeutic targets of lncRNAs in CAVD.

## 1 Introduction

Calcific aortic valve disease (CAVD) is a prevalent heart valve condition, particularly among the elderly, which is associated with high morbidity and mortality rates (1). This disease is associated with significant economic and social burdens. The primary risk factors for CAVD are bicuspid aortic valve (BAV) and advanced age (2). CAVD is characterized by fibrous calcification of the aortic valve leaflets. In the early stages, the disease presents with valve thickening and mild calcification that do not impair the valve function. However, calcification exacerbates over time, resulting in aortic stenosis (AS) (3). Severe AS is often accompanied by shortness of breath, angina, and syncope. Treatment options for severe AS include surgical aortic valve replacement (SAVR) and transcatheter aortic valve replacement (TAVR) (4).

Long noncoding RNAs (lncRNAs) are noncoding RNAs greater than 200 nucleotides in length. As significant and widely used regulatory elements, they play important roles in various cellular processes and development of numerous diseases (5–7). Initially, lncRNAs were thought to be “transcriptional noise,” but they were later recognized to have precise regulatory functions (8). As competitive endogenous RNA (ceRNA), lncRNAs exert gene regulatory functions by sponging miRNAs, directly binding to proteins to alter their function, or serving as scaffolds to recruit transcriptional inhibitors or activators (9, 10). An increasing number of studies have shown that lncRNAs play critical roles in the occurrence and development of cardiovascular diseases, including CAVD, acute myocardial infarction, and heart failure (5, 11). Specific biomarkers may predict calcification progression or disease prognosis, thereby aiding in diagnosis and treating CAVD. This article reviews the research progress regarding lncRNAs in CAVD.

## 2 Pathogenesis of CAVD

Although CAVD pathogenesis is not fully understood, it is believed to result from cellular dysfunction and dysregulation (12). This highly regulated process occurs at the molecular and cellular levels and involves various factors, such as endothelial dysfunction and injury, inflammation, oxidative stress, lipid metabolism disorders, calcium deposition, and extracellular matrix remodeling (12–15). These factors ultimately lead to fibrosis and ossification of valve interstitial cells (VICs), the main cellular components of the aortic valve leaflets (16). Inactive VICs are usually activated and undergo phenotypic transformation into osteoblast-like VICs, which calcify the aortic valves (17). Valvular endothelial cells (VECs) cover the valve surface to form an endothelial monolayer and may undergo endothelial-to-mesenchymal transformation (End-MT), expressing mesenchymal characteristics and plays a pathological role in CAVD development (18, 19). During the occurrence and development stages of CAVD, the abnormal accumulation of different lipid types and increased expression of inflammatory factors such as TNF-α and IL-6 promote osteogenic processes and calcification of VICs (2, 20). Increasing evidence suggests that various signaling pathways such as the NF-κB, TGF-β, Wnt/β-catenin, Notch, and BMP pathways, are involved in aortic valve fibrosis and calcification (21–25). These signaling pathways interact with each other to regulate the occurrence and development of CAVD.

In other cardiovascular diseases, certain lncRNAs have been implicated in the regulation of inflammation, oxidative stress responses and lipid metabolism (26). For example, H19 represses oxidative stress to improve diabetic cardiomyopathy (27), HOTAIR regulates oxidative stress and cardiac myocyte apoptosis during ischemia-reperfusion injury (28), and MALAT1 is involved in the regulation of lipid accumulation and chronic inflammation in atherosclerosis (29). This suggests that lncRNAs may interact with other associated factors to collaboratively modulate the progression of CAVD.

## 3 Roles and molecular mechanisms of lncRNAs in CAVD

### 3.1 HOTAIR

HOX transcript antisense intergenic RNA (HOTAIR) is a 2.2 kb lncRNA believed to regulate HOX expression (30). It can bind to polycomb repressive complex 2 (PRC2) and inhibit the transcription of specific genes by promoting epigenetic modifications of H3K27me3 (30, 31). HOTAIR has received widespread attention as a biomarker for tumor diagnosis (32). Recent studies have shown that HOTAIR plays an important regulatory role in CAVD. Compared to the normal tricuspid aortic valve (TAV), BAV leaflets are subjected to greater mechanical stress, resulting in a significant decrease in HOTAIR expression (33). This decreased expression increases the expression of calcification genes such as ALPL and BMP2, promoting calcification of the aortic valve (33, 34). The WNT/β-catenin signaling pathway plays a crucial role in this process, and mechanical stress can activate this signaling pathway, inhibiting the expression of HOTAIR (33). However, further studies are needed to determine whether HOTAIR directly regulates CAVD.

### 3.2 H19

H19 is another lncRNA involved in CAVD. Its mature product has a total length of 2.3 kb, does not encode proteins but can act on multiple gene regulatory levels to exert its biological functions (35, 36). H19 is the first imprinted gene to be discovered and shows parent-of-origin epigenetic signatures (37).

Hadji et al. found that the expression of H19 is significantly increased in calcified aortic valves and can be upregulated in the early stages of the disease (38). Its expression level is positively correlated with the progression rate of aortic valve stenosis. In CAVD tissue, the CpG methylation level in the H19 promoter region is significantly reduced, which is the main reason for the significant increase in H19 expression. Osteogenic genes, such as RUNX2, bone morphogenetic protein-2 (BMP2), and osteocalcin (BGLAP) are involved in calcification (39–41). *In vitro* experiments have shown that H19 overexpression can promote the BMP2 and RUNX2 expression, as well as the osteogenic phenotype, in VICs (38). Notch1 signaling is an important regulatory pathway for the transdifferentiation of VICs into osteoblast-like cells (42, 43). H19 can inhibit the binding of transcription factor p53 to the promoter region of Notch1, upregulating the expression of the calcification-promoting genes BMP2 and RUNX2 by inhibiting the Notch1 signaling pathway and promoting the occurrence of CAVD (38).

### 3.3 MALAT1

The metastasis-associated lung adenocarcinoma transcript 1 (MALAT1) was initially identified in early non-small cell lung cancer. Recently, MALAT1 has been shown to be involved in the pathological regulation of cardiovascular diseases at various levels (29).

Xiao et al. compared the MALAT1 expression levels in calcified and adjacent normal tissues in 20 pairs of calcified aortic valves (44). They found, for the first time, that MALAT1 expression is significantly increased in the calcified leaflet tissue and shows a gradually increasing trend after osteogenic induction in VICs. The expression levels of MALAT1 and osteogenesis-specific genes such as ALP and osteocalcin are highly positively correlated. Further research has found that MALAT1 overexpression can promote the osteogenic differentiation of VICs, whereas silencing MALAT1 can inhibit ALP activity induced by the osteogenic medium, calcified nodule formation, and osteocalcin expression. They also found that MALAT1 sponges miR-204, thereby inhibiting its expression and activity. Smad4 is a direct target of miR-204. Overexpression of MALAT1 upregulates Smad4 by inhibiting miR-204 expression, thereby promoting the osteogenic differentiation of VICs.

### 3.4 TUG1

Taurine-upregulated gene 1 (TUG1) is located on chromosome 22q12 and is approximately 7.1 kb in length (45). As a competitive endogenous RNA (ceRNA), it regulates gene expression by sponging miRNAs and is involved in the development of various cancers (46). Increasing evidence indicates that TUG1 is involved in the development and prognosis of cardiovascular diseases (47).

Yu et al. have reported the mechanism of action of TUG1 in CAVD (48). They compared the expression levels of TUG1 in 40 pairs of calcified aortic valve and adjacent normal valve tissues and found that its expression is significantly increased in calcified valve tissues. *In vitro* cell experiments have shown that TUG1 is significantly upregulated during the calcification of VICs and is positively correlated with the expression levels of osteogenic markers, including ALP, osteocalcin, and osteopontin. Silencing TUG1 can inhibit the differentiation of VICs into osteoblast-like cells and calcified nodule formation, whereas its overexpression leads to the opposite result. The study also found that TUG1 interacts with miR-204-5p and promote RUNX2 expression by regulating miR-204-5p. Additionally, the regulatory effect of TUG1 on aortic valve calcification was confirmed in an ApoE^−/−^ mouse model. They also found that targeting of TUG1 significantly reduces high-cholesterol diet-induced aortic valve calcification in ApoE^−/−^ mice, which can provide evidence for a therapeutic strategy in CAVD treatment.

### 3.5 OIP5-AS1

OIP5 antisense transcript 1 (OIP5-AS1) is highly expressed in the nervous system and plays a crucial role in tumor transformation (49). OIP5-AS1 also plays a regulatory role in heart diseases. Hadji et al. performed RNA sequencing on 9 tricuspid valve CAVDs and 10 control non-calcified aortic valves and found that, compared with the control non-calcified aortic valves, OIP5-AS1 is significantly downregulated in calcified aortic valves (38). Zheng et al. conducted further research on its regulatory mechanisms (50). *In vitro* experiments have shown that during the osteogenic differentiation of VICs, the expression of OIP5-AS1 is significantly reduced, whereas the mRNA levels of osteogenic differentiation markers, including ALP, osteocalcin, and osteopontin, are increased. In addition, the overexpression of OIP5-AS1 in VICs significantly decreased the levels of these osteogenic differentiation markers. Mechanistically, further experiments showed that OIP5-AS1 could alleviate the osteogenic differentiation of VICs by upregulating TWIST1, the target gene of miR-137.

### 3.6 AFAP1-AS1

Actin filament-associated protein 1 antisense RNA 1 (AFAP1-AS1) is a 6810-nt lncRNA transcribed from the antisense DNA strand of *AFAP1* (51). It is upregulated in various tumors and is associated with tumorigenesis (52).

In terms of heart disease, Hadji et al. found that AFAP1-AS1 level in calcified aortic valves is significantly higher than those in non-calcified aortic valves (38). Further research by He et al. showed that the expression level of AFAP1-AS1 significantly increased after calcification of the aortic valves and osteogenic induction of human VICs (53). Additionally, overexpression of AFAP1-AS1 promoted the osteogenic differentiation of VICs, whereas knockdown of AFAP1-AS1 inhibited osteogenic differentiation. Mechanistically, AFAP1-AS1 can sponge miR-155, thereby increasing the expression of SMAD5 to promote the osteogenic differentiation of VICs.

Moreover, compared to normal aortic valves, calcified ones are often accompanied by a large amount of macrophage infiltration, and the number of macrophages is positively correlated with the degree of calcification and diseases severity (54–56). In addition, studies by He et al. have shown that AFAP1-AS1 can aggravate the osteogenic differentiation of VICs by promoting the polarization of M1 macrophages, playing an important role in CAVD (56).

### 3.7 SNHG3

Small nucleolar RNA host gene 3 (SNHG3) is located on chromosome 1p35 and is mainly associated with the occurrence and development of various malignant tumors (57). Chen et al. found that SNHG3 is highly expressed in the calcified valves of patients with CAVD and in VICs undergoing osteogenic differentiation, and that SNHG3 expression is positively correlated with CAVD progression (58). Silencing of SNHG3 can inhibit ALP activity induced by the osteogenic differentiation medium, calcium ion concentration, calcified nodule formation, and protein levels of osteogenic differentiation markers. Conversely, SNHG3 overexpression promotes the osteogenic differentiation of VICs. *In vivo* experiments show that SNHG3 silencing ameliorates aortic valve calcification independent of metabolic regulation in ApoE^−/−^ mice. Further research demonstrated that SNHG3 inhibits trimethylation of the BMP2 promoter by interacting with EZH2 (the core component of PRC2) and activates the BMP2 signaling pathway, thereby promoting the osteogenic differentiation of VICs.

### 3.8 FGD5-AS1

FGD5 antisense RNA 1 (FGD5-AS1) is a newly discovered lncRNA that plays an important role in cardiovascular diseases, including congenital heart diseases, myocardial ischemia/reperfusion injury, acute myocardial infarction, and dilated cardiomyopathy (59–62). Recently, Wei et al. found that the expression levels of FGD5-AS1 and BIRC5 are decreased in patients with CAVD, whereas the expression level of miR-497-5p increased (63). *In vitro* cell experiments demonstrated that osteogenic differentiation of VICs leads to increased ALP activity and calcium nodules, with gradually increasing levels of osteogenic differentiation markers (RUNX2 and OPN). Subsequent results showed that overexpression of FGD5-AS1 results in decreased ALP activity and calcium nodules, as well as expression of RUNX2 and OPN, thereby alleviating CAVD. Further mechanistic studies revealed that FGD5-AS1 regulates the osteogenic differentiation of VICs through the miR-497-5p/BIRC5 pathway. *In vivo* experiments have shown that enhancement of FGD5-AS1 effectively alleviates aortic valve leaflet thickness and calcium deposition in ApoE^−/−^ mice (Table 1).

**Table 1:** Mechanisms of lncRNAs in CAVD.

LncRNAs	Mechanism	Targets	Species	Ref
LncRNA HOTAIR	Down	–	BAV vs. TAV	(33)
LncRNA H19	Up	NOTCH1	CAVD vs. HT aortic valve	(38)
LncRNA MALAT1	Up	miR-204/SMAD4	CAVD (calcified aortic valves vs. adjacent normal tissues)	(44)
LncRNA TUG1	Up	miR-204-5p/Runx2	CAVD (calcified aortic valves vs. adjacent normal tissues)	(48)
LncRNA OIP5-AS1	Down	miR-137/TWIST11	VICs isolated from normal aortic valves	(50)
LncRNA AFAP1-AS1	Up	miR-155/SMAD5	DCAVD (calcified aortic valves vs. adjacent normal tissues)	(53)
LncRNA SNHG3	Up	BMP2 pathway	CAVD vs. HT aortic valve	(58)
LncRNA FGD5-AS1	Down	miR-497-5p/BIRC5	CAVD vs. AI aortic valve	(63)

BAV, bicuspid aortic valve; TAV, tricuspid aortic valve; CAVD, calcific aortic valve disease; HT, heart transplantation; VICs, valve interstitial cells; DCAVD, degenerative calcific aortic valve disease; AI, aortic valve insufficiency.

## 4 Treatment of CAVD

Currently, apart from TAVR and SAVR, no effective drug treatment is available to prevent or slow CAVD progression (4, 64, 65). Identifying sensitive and specific biomarkers like lncRNAs for the early detection of CAVD is crucial. Early detection could enable the timely application of therapeutic interventions, potentially slowing or halting the progression of CAVD and preventing irreversible damage to the aortic valve (24). Studies have demonstrated that the repression of TUG1 and SNHG3, as well as enhancement of FGD5-AS1, can ameliorate aortic valve calcification in mice, offering potential therapeutic avenues for lncRNA-based treatment of CAVD (48, 58, 63).

Although no treatments directly targeting lncRNA have yet reached clinical application, their therapeutic potential is incontestable (66). The approaches to lncRNA targeting such as antisense oligonucleotides (ASOs), small interfering RNAs (siRNAs), and CRISPR-Cas9 may become novel treatment strategies for CAVD (67). These techniques are frequently employed in cancer research and treatment. For example, ASOs targeting MALAT1 have been shown to effectively reduce tumor growth and metastasis in lung cancer (68). Silencing HOTAIR through RNA interference (RNAi) inhibits the invasion and proliferation of human colon cancer cells (69). The CRISPR-Cas9 system facilitates targeted gene editing by generating double-strand breaks (DSBs) in the DNA (70). Several approaches have been developed to manipulate lncRNA expression using the CRISPR-Cas9 system, including CRISPR interference (CRISPRi), CRISPR activation (CRISPRa), and CRISPR-Cas9 mediated knockout (70). Targeting MALAT1 by CRISPR/Cas9 technique inhibits the cell proliferation and migration in prostate cancer (71). Targeting lncRNAs with the CRISPR-Cas9 system may be a promising strategy for developing novel therapeutic interventions.

The delivery system is crucial for the therapeutic potential of lncRNAs. It must exhibit satisfactory specificity, stability, cell permeability, and low immunogenicity (72). Nanoformulation-mediated delivery and exosome-mediated delivery of targeted lncRNAs are promising avenues (73). A study reveals the therapeutic potential of lipid nanoparticles (LNPs) for targeted OIP5-AS1 delivery in mitigating MI/R injury (74). In another study, it was demonstrated that exosomes containing oxaliplatin and lncRNA PGM5-AS1 can reverse oxaliplatin resistance in colorectal cancer therapy (75). And exosome-mediated delivery of CRISPR-Cas9 has been identified as a biocompatible delivery system for gene editing in oncology (76).

However, the development of lncRNA-targeted therapeutics for CAVD will encounter challenges such as off-target effects, delivery specificity and stability. Modifications of Cas9 or sgRNA, along with sgRNA truncation, are effective strategies to reduce off-target effects (77–79). The chemical modification of ASOs may overcome the challenges associated with poor stability (72). And before proceeding to clinical trials, safety and toxicity studies must be conducted. Therefore, further technological progress is required.

## 5 Discussion

CAVD is a common heart valve disease characterized by multifactorial and multistep pathological processes including endothelial dysfunction, inflammation, lipid metabolism disorders, calcium deposition, and other complex biological mechanisms (12). Currently, the research significance of lncRNAs has increased, and growing attention has been focused on relationship between CAVD and lncRNAs. As biomarkers, lncRNAs have certain advantages. In a study published in Science in 2017, 499 lncRNA molecules with important functions for cell growth were identified, of which 89% functioned in specific cell types, indicating that lncRNA has strong specificity (80). And lncRNAs can be detected in the early stages of the disease, offering a potential basis for early diagnosis (38). In addition, the stability of lncRNA in the bloodstream makes it an ideal candidate for non-invasive biomarkers (81).

In recent years, studies have gradually revealed the roles of lncRNAs in regulating gene expression in CAVD. Some studies have confirmed that lncRNAs regulate the osteogenic differentiation of VICs or participate in valve calcification by sponging miRNAs. However, the regulatory mechanisms of lncRNAs are complex, and there may be multiple regulatory modes. For example, some researchers have speculated that HOTAIR may improve CAVD by sponging miR-29b (82), whereas MALAT1 may exacerbate CAVD by sponging miR-195 (83). Additionally, MALAT1 participates in osteogenic differentiation through MAPK and Wnt/β-catenin signaling pathways in osteoporosis (84, 85) and H19 induces an osteogenic phenotype in isolated human mesenchymal stem cells via the Wnt pathway (86), indicating that they may also be involved in aortic valve calcification. However, the proposed mechanism requires further experimental validation.

In addition to the previously mentioned lncRNAs that have regulatory effects on CAVD, other lncRNAs, such as myocardial infarction-associated transcript (MIAT) and antisense non-coding RNA in the INK4 locus (ANRIL), may regulate osteogenic differentiation and inflammatory responses in various cells, suggesting that they could have similar mechanisms in the aortic valve. MIAT is closely related to cardiovascular disease and is involved in the NF-κB pathway, promoting the expression of pro-inflammatory cytokines like IL-6 and TNF-α, and influencing the osteogenic differentiation of human adipose-derived and bone marrow mesenchymal stem cells (87–92). ANRIL, associated with atherosclerosis, regulates osteogenic differentiation in periodontal ligament and stem cells, and may also be involved in the Wnt/β-catenin and NF-κB signaling pathways (93–97). Therefore, both MIAT and ANRIL may play a role in the valve calcification seen in CAVD; however, further research is needed to clarify their specific contributions. We can identify novel lncRNAs through high-throughput RNA sequencing and profiling as well as bioinformatic analysis.

In summary, lncRNAs play crucial roles in the occurrence and development of CAVD. The mechanisms of action of lncRNAs in CAVD are complex and not fully understood, complicating their translation to therapeutics. Further investigation of their mechanisms of action will help elucidate the pathological processes underlying CAVD and provide new insights to enable early diagnosis and targeted therapy.
